# A simultaneous bilateral asymmetric hip fracture in an elderly patient: A case report and review of the literature

**DOI:** 10.1016/j.ijscr.2020.06.031

**Published:** 2020-06-12

**Authors:** Kristian Efremov, Alessandro Caterini, Fernando De Maio, Pasquale Farsetti

**Affiliations:** Department of Orthopaedic Surgery, University of Tor Vergata, Viale Oxford 81, 00133 Rome, Italy

**Keywords:** Bilateral hip fracture, Simultaneous, Intramedullary nail, Hemiarthroplasty, Case report

## Abstract

**Introduction:**

Simultaneous bilateral hip fractures are usually associated with high energy trauma, seizures, metabolic diseases and bisphosphonates use. They are observed rarely in the elderly population after simple trauma and usually have the same fracture pattern (symmetric). We report a rare case of asymmetric fracture and discuss mechanism of injury and treatment, analyzing the literature.

**Presentation of case:**

We report a case of an 86-year old woman with a simultaneous bilateral asymmetric hip fracture (trochanteric on the right side and subcapital on the left), occurred after a fall at home. The patient was surgically treated by open reduction and internal fixation with an intramedullary nail on the right and by hemiarthroplasty on the left performed during the same surgical session, with good results.

**Discussion:**

Simultaneous bilateral hip fractures in the elderly are rare and usually are symmetric. In the majority of the cases these fractures are intracapsular and are treated by hemiarthroplasties. On the contrary, simultaneous bilateral hip fractures with a different pattern, as the described case, are extremely rare. The possible mechanism of injury is a spontaneous fracture on one side, which causes a fall the contralateral side. The surgical treatment may be performed bilaterally, treating first the trochanteric fracture.

**Conclusion:**

Simultaneous bilateral asymmetric hip fracture are extremely rare and may occur in elderly patient with a specific mechanism of injury. We believe that the trochanteric fracture should be operated first to avoid possible complications.

## Highlights

• Simultaneous bilateral hip fractures in the elderly are rare and usually have a symmetric pattern.
• Intracapsular fractures on one side and trochanteric on the other are even rarer.
• They are caused by a spontaneous fracture which causes a fall on the contralateral side.
• The trochanteric fracture should be operated first to avoid possible complications.

## 1 Introduction

Hip fractures, including intracapsular and trochanteric fractures, represent an important healthcare problem in the entire world due to its high incidence, mortality, and global healthcare cost [1]. Patients with hip fracture have a risk of a new fracture on the contralateral side that ranges between 9% and 14% [2,3]. Most of the contralateral fractures that occur following the first fracture, appear to have the same pattern of the original one; some authors [2] reported a series of 241 non simultaneous bilateral hip fractures in which 81% of patients had the same type of fracture and treatment.

Simultaneous bilateral hip fractures, on the contrary, are quite rare, especially in relationship to the very high incidence of unilateral fractures, that keeps increasing as the population ages. Simultaneous bilateral fractures are more frequent in younger patients due to high energy trauma [4], seizures or contractions following electroconvulsive therapy [5] and metabolic disease [6]. Other causes for these fractures are the use of bisphosphonates [7] or stress fractures [8]. More rarely, simultaneous bilateral hip fractures are also present in the elderly patients due to low energy trauma, generally in patients affected by osteoporosis [[9], [10], [11], [12], [13], [14], [15], [16], [17], [18], [19]]. Usually, simultaneous hip fractures have the same pattern (intracapsular or trochanteric) and require the same treatment bilaterally, while simultaneous hip fracture with asymmetric pattern are extremely rare.

We report a case of a simultaneous bilateral asymmetric hip fracture in an elderly patient after a minimal trauma, with an intracapsular fracture on one side and a trochanteric fracture on the other.

## 2 Presentation of case

This paper was reported in line with the SCARE 2018 criteria [20].

An 86-year-old female was admitted to the emergency department of our hospital following a fall at home. The patient reported feeling sudden pain in her left hip but still managed to stand upright then fell down on the other side. History revealed a colorectal carcinoma surgically treated several years earlier, and a previous thyroidectomy for unknown reason. Family and psychosocial history were not relevant. She did not take any chronic medical therapy, but did appear malnourished. She complained of bilateral hip pain more severe on the right, with a shortened and externally rotated right limb. The left limb had no deformities but was painful to palpation and during passive motion in the inguinal region.

**Fig. 1:**
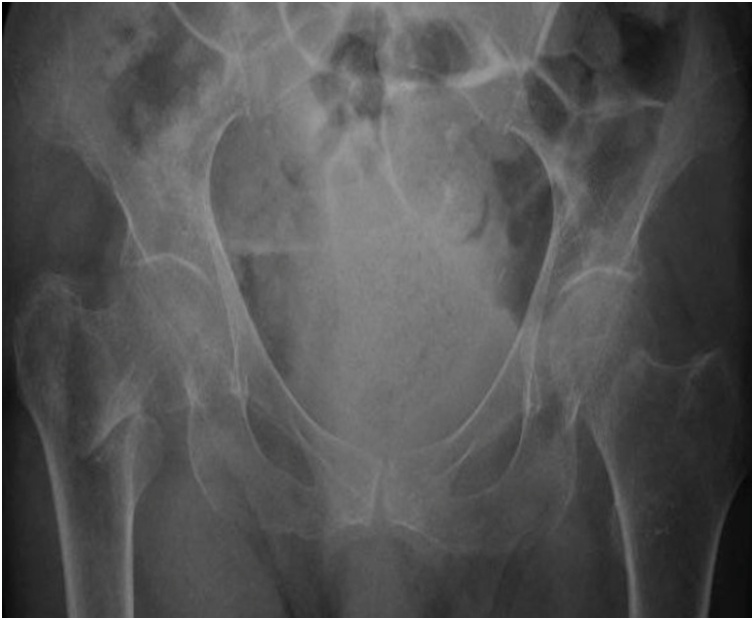
Preoperative radiographic examination of both hips in AP view of a patient with bilateral simultaneous hip fracture with different pattern showed a trochanteric fracture of the right and a subcapital fracture of the left.

**Fig. 2:**
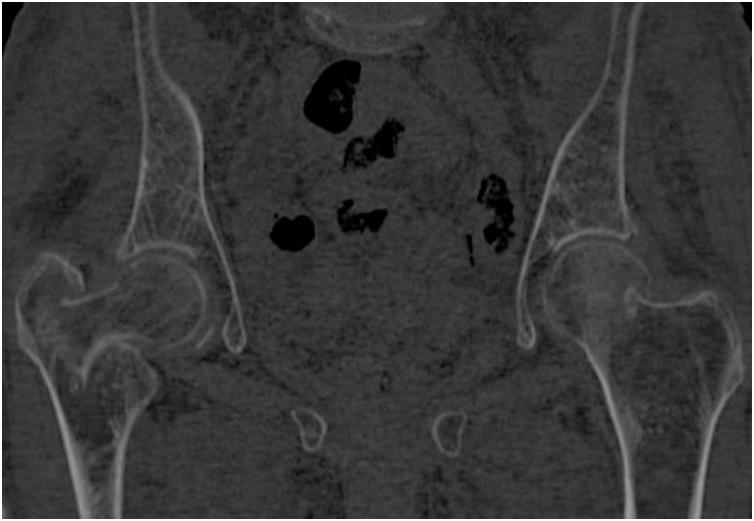
Preoperative CT scan of both hips confirmed the radiographic diagnosis and excluded pathological fractures.

**Fig. 3:**
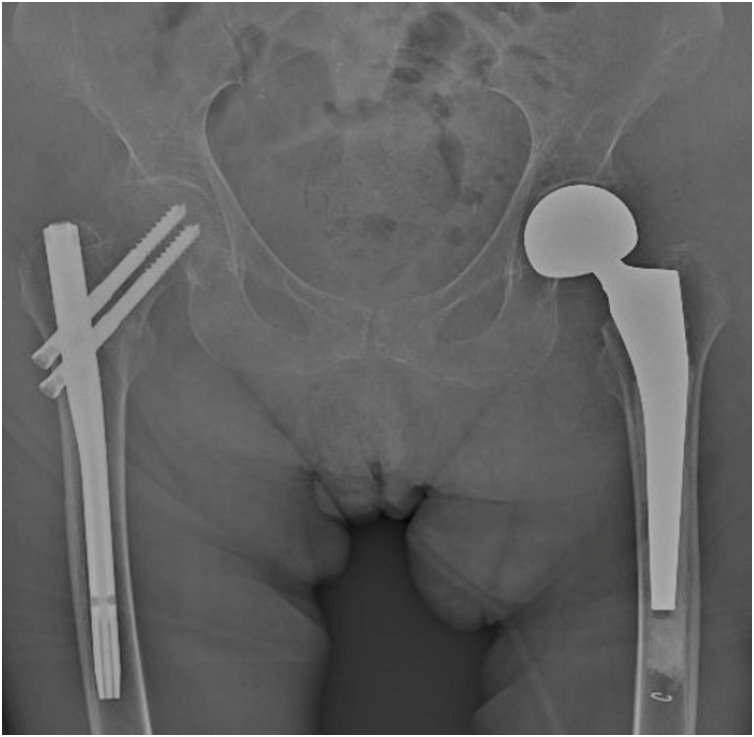
Postoperative radiographic examination of the hip in AP view of the same patient surgically treated by ORIF with an intramedullary nail on the right and by hemiarthroplasty on the left.

The patient underwent a pelvic radiographic examination followed by a CT scan which showed a trochanteric fracture of the right femur and a subcapital fracture on the left (Figs. 1, 2). The CT scan showed no evidence of pathological fractures (Fig. 2). The patient was brought in the operating room the next day where, on a traction table, we first performed reduction and internal fixation of the right femur with an intramedullary nail (Citieffe Standard 195 mm, blocked with two cephalic screws without a distal lock). Afterwards, the patient was positioned in lateral decubitus, and underwent cemented biarticular hemiarthroplasty (Permedical). The procedure was performed by the senior author (PF). The total surgical time was 2 h and 20 min. The post-operative radiographic examination showed good reduction and stabilization of the right femur and a well-positioned hemiarthroplasty on the left (Fig. 3).

The patient required two units packed red blood cells on the first post-operative day (Hb: 7.5 g/dl). The same day she started physical therapy and on the seventh post-operative day she was transferred to a rehabilitation clinic to continue physical therapy, which was completed successfully in two months. Physical therapy was longer than usual because both hips were involved.

At final follow up, 6 months after surgery, the patient was pain free, able to walk autonomously with good range of motion of both hips. She was satisfied with the final result.

## 3 Discussion

Hip fractures are an important social problem due to the high incidence, morbidity, mortality and cost. In a large epidemiologic study, the incidence of intracapsular and extracapsular fractures is similar [21]. Frequently hip fractures can be bilateral, but occurring in two different times, with the same fracture pattern [2]. The reason of this phenomenon may be due to the symmetry that each patient has in their own gait and bone femoral neck architecture [2].

On the contrary, bilateral simultaneous hip fractures are rare, and often occur in younger patients caused by high energy trauma [4], seizures [5] and metabolic disease [6]. These fractures are also reported in patients treated by bisphosphonates, and usually are atypical sub-trochanteric fractures [7].

Bilateral simultaneous hip fractures are even less common in elderly patients without significant comorbidities when caused by trivial trauma and few cases are reported in the literature [[9], [10], [11], [12], [13], [14], [15], [16], [17], [18], [19]] (Table 1). Almost all of the described cases have a symmetric fracture pattern and underwent the same treatment bilaterally (arthroplasty for intracapsular fractures and ORIF by intramedullary nail for extracapsular fractures) [[10], [11], [12], [13],[15], [16], [17], [18], [19]].

**Table 1:** Simultaneous bilateral hip fracture in elderly patients: review of the literature.

Author	Year of publication	Case	Age	Sex	Mechanism of trauma	Type of fracture	Treatment
Robin [9]	1963	1	87	M	n/a	Intracapsular and extracapsular	Hemiarthroplasty + ORIF by nail-plate
Kumar et al. [10]	1997	1	89	F	Tripped and fell	Bilateral intracapsular	Bilateral Moore hemiarthroplasty
Carpintero et al. [11]	2006	1	77	M	Fall after acute pain on one side	Bilateral intracapsular	Died before surgery
		2	81	M	Fall after acute pain on one side	Bilateral intracapsular	Bilateral Thompson hemiarthroplasty
Grisoni et al. [12]	2008	1	88	F	Same level fall	Bilateral extracapsular	Bilateral ORIF by dynamic hip-screws
Sood et al. [13]	2009	1	84	M	Simple fall	Bilateral intracapsular	Bilateral Thompson hemiarthroplasty
Park et al. [14]	2015	1	83	F	Simple fall	Bilateral intracapsular	In situ pinning and bipolar hemiarthroplasty
Aydın et al. [15]	2015	1	76	M	Slipped and fell	Bilateral extracapsular	Bilateral ORIF by intramedullary nail
van der Zeeuw et al. [16]	2016	1	90	M	Fall from bed	Bilateral extracapsular	Bilateral OROF by intramedullary nail
Popescu et al. [17]	2016	1	90	F	Same level fall	Bilateral intracapsular	Bilateral uncemented bipolar hemiarthroplasties
Vijayvargiya et al. [18]	2016	1	66	F	Fall at home	Bilateral intracapsular	Bilateral cemented total hip arthroplasty
McDonald et al. [19]	2019	1	89	M	Fall at after legs gave away	Bilateral intracapsular	Bilateral Thompson hemiarthroplasty

From the 11 selected studies in which the authors reported 12 cases of elderly patients (age range: 66–90 years), 8 had a bilateral simultaneous intracapsular fracture [10,11,13,14,17,19], while 3 had an extracapsular fracture [12,15,16]. All these fractures were symmetric with the same fracture pattern.

Regarding the mechanism of trauma of the intracapsular fractures, all were caused by simple falls; in two cases it was specified that the patients reported acute pain on one side before falling down while one patient fell after his legs gave away. Regarding the treatment, 5 patients underwent bilateral hemiarthroplasty (Thompson in three cases, Moore in one and uncemented bipolar hemiarthroplasty in the last); one patient underwent a bilateral cemented total hip arthroplasty, one had a bipolar hemiarthroplasty in one side and in situ fixation with cannulated screw in the other and one patient died before surgery.

Regarding the mechanism of trauma of the extracapsular fractures, all were caused by simple falls, one of whom fell while trying to get out of bed. Regarding the treatment, all three patients underwent reduction and internal fixation (two patients with bilateral intramedullary nails and one patient with bilateral dynamic hip screws).

Only one patient had an asymmetric fracture; the patient had an intracapsular fracture on the left and a trochanteric fracture on the right. The mechanism of injury was unknown. The intracapsular fracture was treated by a Moore hemiarthroplasty, while the trochanteric fracture was stabilized with a McLaughlin nail-plate. Both surgical procedures were performed in a prone position [9].

To the best of our knowledge, our case represents only the second case of simultaneous, bilateral asymmetric hip fracture. Regarding the mechanism of injury, according to the clinical history, we believe that the patient initially had the subcapital fracture which caused acute left groin pain, that led to a fall with trauma of the right hip, causing the contralateral trochanteric fracture. Therefore, we believe that in addition to the importance of bone architecture mentioned by other authors [2] the mechanism of injury may play a role in determining the fracture pattern.

Regarding the sequence of the surgical treatment, preoperative planning is important. We decided to treat the trochanteric fracture first, performed on a traction table, and stabilized by intramedullary nail after reduction. Afterwards, we performed a hemiarthroplasty on the contralateral side in the lateral decubitus. We opted for this sequence for two reasons. First, we believe that it is easier to switch the patient from the traction table to a lateral decubitus than viceversa; second, to avoid the risk of dislocation of the prosthesis when positioning the patient on the traction table.

## 4 Conclusion

In conclusion, the simultaneous bilateral asymmetric hip fractures are extremely rare and may occur also in elderly patient with a specific mechanism of injury. We believe that the trochanteric fracture should be operated first to avoid possible complications, such as prosthesis dislocation, which can occur when positioning the patient on the traction table.

## Sources of funding for your research

None.

## Ethical approval

None.

## Consent

Written informed consent was obtained from the patient for publication of this case report and accompanying images.

## Registration of research studies

NA.

## Guarantor

Prof. Pasquale Farsetti.

## Provenance and peer review

Not commissioned, externally peer-reviewed.

## CRediT authorship contribution statement

**Kristian Efremov:** Conceptualization, Writing - original draft. **Alessandro Caterini:** Investigation. **Fernando De Maio:** Writing - review & editing. **Pasquale Farsetti:** Writing - review & editing, Supervision.

## Declaration of Competing Interest

We certify that no benefits in any form have been received or will be received from a commercial party related to the subject of this article. No funds were received in support of this study.
